# Population Grids for Analysing Long-Term Change in Ethnic Diversity and Segregation

**DOI:** 10.1007/s40980-020-00071-6

**Published:** 2020-12-21

**Authors:** Gemma Catney, Christopher D. Lloyd

**Affiliations:** Queen’s University Belfast, Geography, School of Natural And Built Environment, Belfast, BT7 1NN UK

## Abstract

Changes in the spatial patterns of ethnic diversity and residential segregation are often highly localized, but inconsistencies in geographical data units across different time points limit their exploration. In this paper, we argue that, while they are often over-looked, population grids provide an effective means for the study of long-term fine-scale changes. Gridded data represent population structures: there are gaps where there are no people, and they are not (unlike standard zones) based on population distributions at any one time point. This paper uses an innovative resource, *PopChange*, which provides spatially fine-grained (1 km by 1 km) gridded data on country of birth (1971–2011) and ethnic group (1991–2011). These data enable insight into micro-level change across a long time period. Exploring forty years of change over five time points, measures of residential ethnic diversity and segregation are employed here to create a comprehensive ‘atlas’ of ethnic neighbourhood change across the whole of Britain. Four key messages are offered: (1) as Britain’s ethnic diversity has grown, the spatial complexity of this diversity has also increased, with greater diversity in previously less diverse spaces; (2) ethnic residential segregation has steadily declined at this micro-scale; (3) as neighbourhoods have become more diverse, they have become more spatially integrated; (4) across the whole study period, the most dynamic period of change was between 2001 and 2011. While concentrating on Britain as a case study, the paper explores the potential offered by gridded data, and the methods proposed to analyse them, for future allied studies within and outside this study area.

## Introduction

Analyses of neighbourhood change are often subject to compromises around the size of spatial units and temporal inconsistencies in their boundaries (Martin et al. 2002). These limitations are particularly significant in ethnic and racial studies, where spatial patterns, and alterations to them, may be highly localized (e.g., Reardon et al. 2008; Östh et al. 2015; Johnston et al. 2016; Barros and Feitosa 2018; Catney 2018; Ellis et al. 2018; Yao et al. 2019; Lan et al. 2020; Catney et al. 2020). Administrative changes in the size and shape of spatial units (zones) used to report data from Censuses are implemented to respond to local-level population growth or decline. Because zones are thus often inconsistent between time points, spatial units must be standardized for analyses of change over time. Most approaches transfer population data from zones at one (or more) time point(s) to make them consistent with the most recent set of zones used in the analysis. This means that the results for earlier time points are based on zones designed with a different population structure (for example, 1991 data using 2011 boundaries). Using the most contemporary geographical areas for comparisons over time is logical, and commonly applied; examples include Norman (2016) for deprivation from 1971 to 2011, and Catney (2016a) for ethnic diversity from 1991 to 2011. In this paper, we argue that population grids offer an important alternative solution to approaches based on the population structure for a single time point. We exploit an innovative Census-based gridded dataset, *PopChange*—a freely-available resource comprising harmonised variables and consistent small areas for the whole of Great Britain (England, Scotland and Wales) from 1971 to 2011 (Lloyd et al. 2017).^1^ We use *PopChange* data on country of birth (1971–2011) and ethnic group (1991–2011) to explore changes in Britain’s ethnic residential geographies at a more micro-scale and longer time period than has hitherto been possible. Utilising 1 by 1 km grids over forty years and five time points, we showcase a novel approach to understanding the changing spatial patterns of ethnic groups. We illustrate the utility of this resource for the British case, but also make a broader contribution in demonstrating the research potential of temporally rich population grids, for future allied studies within and outside this study area.

The period that is the subject of our analysis transects a time of diversifying immigration streams, growing and maturing UK-born ethnic minority populations, and new patterns of internal migration. The growth of ethnic diversity in the UK has been accompanied by intense interest in the changing geographies of racialised minority groups. Much of the scholarship in this area has emphasised the significance of small areas—neighbourhoods—in understanding the drivers, and resultant patterns, of (ethnic) demographic change (Johnston et al. 2016; Catney 2018; Lan et al. 2020). Yet despite this interest in the local, and on temporal trends, a British ‘atlas’ documenting changing ethnic geographies has not yet been attempted at this scale, and for such a long time period. What can we learn about the changing micro-geographies of ethnicity from a longitudinal study of small areas? Gridded data enable the temporal exploration of the extent and spatial scales of diversity on a consistent basis. Exploiting these proprieties, we show how diversity has evolved within and between areas, and over what scales—from the local, to the regional. We are also able to identify which period saw the most marked changes in diversity and segregation; when has Britain’s neighbourhood change been most dynamic? Since *PopChange* provides consistent data for the whole of Great Britain, we are able to explore the rapidly shifting ethnic geographies of Scotland, which has received considerably less attention given issues of data comparability. Our analytical strategy is novel in making use of grids, and in applying a suite of methods in their analysis. These include variograms, a method rarely applied in population studies, which, in this paper, is used to provide a quantitative summary of the spatial extent of diversity and how this has changed over a long time period.

We next consider some of the benefits of population grids over the use of standard output units in analyses of ethnic diversity and residential segregation, before we provide a necessarily concise summary of the rich history of the origins of diversity in Britain since the 1970s. The paper then introduces the *PopChange* resource and describes the country of birth and ethnic group categories used in our analysis. The gridded data are then interrogated using a wide-ranging set of methods to explore the changing geographies of ethnicity in Britain. Each method is introduced in turn, alongside the presentation of results, before finally summarising the findings and reflecting on their implications.

## Population Grids

All zonal systems (such as census wards or tracts) are arbitrary; they also tend to be shaped by population characteristics (e.g., density) at a particular point in time. Further, most standard sets of zones cover all of the area of interest, such that there may be a large rural zone which is essentially ‘empty’, with all residents concentrated within a few small areas of that larger zone. The arbitrary nature of zones cannot be fully overcome, although there have been attempts to derive flexible neighbourhood spaces in place of fixed zones. Lee et al. (2019: 1) suggest the use of “a series of nested local environments surrounding each individual that approximate meaningful domains of experience”, for measuring ethnic diversity. The other limitations listed can be removed through the use of population grids, which may be invariant across time, not dependent on the population structure at any particular time point, and which may have ‘holes’ where there are no people or where population density is low. The presence of gaps is especially significant for analyses of ethnic residential segregation. Standard spatial units cover the whole of the study area, and boundaries between adjacent zones imply that the zones are neighbours. In practice, this may not be the case; population centres in neighbouring zones may be distant from one another, and not located close to their zone boundary. Thus, the capacity for interaction between people in these adjacent zones may be exaggerated. With population grids, the distribution of people *within* standard zones can be taken into account. Another distinct advantage for studies of diversity and segregation is that the spatial scale of observation is constant across all areas—from the most densely populated urban areas, through to sparsely populated rural locales. On the basis that likely interactions are impacted more by distance than by population density, zones of constant size provide a logical basis for comparisons between areas.

The use of population grids is growing in popularity—several European countries publish population statistics using grids (e.g., Finland, the Netherlands, Sweden), and the European Commission, via Eurostat, has produced a population grid called *Geostat* for member countries (Batista e Silva et al. 2013).^2^ One key additional benefit of gridded data is that they more easily facilitate comparisons between countries. Andersson et al. (2018; in a collection of papers using the same datasets) aggregated individual-level data to grids in an analysis of segregation by country of birth in Belgium, Denmark, the Netherlands and Sweden. A recent novel development is the creation of a dataset on the population with migrant backgrounds in EU member states, for 100 m grid cells (Alessandrini et al. 2017). These data have been used to compare the residential segregation of migrant populations in multiple urban areas across Europe (Benassi et al. 2020), and to explore multi-scale segregation in Paris (Olteanu et al. 2020). Other studies using gridded data to explore ethnicity include Wong et al. (1999), who allocate standard zones to grids based on the zone centroids in an analysis of segregation in US cities. Sui and Wu (2006) transferred data from census tracts to grids in a multiscale analysis of segregation in Houston. Shuttleworth and Lloyd (2009) use gridded data for Northern Ireland to assess changing patterns of segregation by religion. Grid cells subdivide larger areas with lower population densities, and thus country-wide statistics based on grids emphasize (relative to standard sets of zones) locations with lower population densities. This has particular advantages for measuring diversity by country of origin and/or ethnic group as it removes the urban bias to which most analyses of standard zones are subject. Thus, grids offer some exciting possibilities for understanding the changing geographies of diversity, which may be spreading outside of metropolitan areas (for example, see Catney 2016a), and they provide a powerful complement to analyses based on standard zonal systems. There is an extensive literature on the spatial scales of residential segregation, and their changes over time (e.g., Reardon et al. 2009; Fowler 2016; Johnston et al. 2016), albeit less so for diversity (exceptions include Wright et al. 2014; Ellis et al. 2018). Despite this, the unique opportunities offered by population grids for analyses of long-term change have been overlooked.

In the UK, gridded data (1 km grid cells for all areas, 100 m grid cells in urban areas) were released as an output from the 1971 Census (see Rhind 1975), but this practice did not continue (largely on the grounds of cost, as discussed by Denham and Rhind 1983), with the exception of Northern Ireland, where grids have been released as outputs from all Censuses since 1971 [see Shuttleworth and Lloyd (2009) for more details]. The 1971 grid data were used to produce a *Census Atlas of Britain* (CRU/OPCS/GROS, 1980). Openshaw and Mounsey (1987) used data from the 1981 Census to generate the Domesday 1986 population grid dataset. There have been a range of subsequent initiatives to produce population grids in Britain, including the work of Martin (1996), Murdock et al. (2015), and Reis et al. (2016). In Britain, population grids are scheduled to be released as outputs from the 2021 Census (2022 in Scotland, on the basis of likely disruption due to the Covid-19 pandemic).

Where geo-referenced household-specific data are available, these can easily be aggregated to grid cells to produce gridded population counts. Statistical agencies in some countries (for example, Finland and Sweden) produce grids in this way (see Batista e Silva et al. 2013 for discussion around European population grids). In other countries where gridded data are not provided, it is necessary to construct population grids by overlaying the standard geographical zones onto a regular grid and reallocating the population counts from the standard zones to the grids. This can be done using an area weighting approach which, at its most simple, entails computing what proportion of each standard unit falls within each grid cell and assigning the relevant proportion of the population to that grid cell. As an example, if 20 percent of a zone falls within a grid cell, then 20 percent of that zone’s population is transferred to that grid cell. More sophisticated approaches entail using landuse data or other ancillary data sources [e.g., postcode locations, as used in the present study and by Norman (2016)] to more accurately transfer counts to grids; Martin (1996) discusses some possible approaches (and see also Lloyd et al. 2017).

## The (Recent) Origins of British Ethnic Diversity and its Geographies

The *PopChange* project starts in 1971, the earliest time point for which small area Census data are available. Coincidentally, this is also the period when immigration flows to the UK were most intensely diversifying. Our story starts at the cessation of the immigration boom of the Windrush generation (1948 to the early 1970s). This inflow of migrants from the Caribbean, alongside other postwar arrivals from, in particular, India (including those from East Africa) and Pakistan (including Bangladesh, formerly East Pakistan) had created new ‘non-White’ diversity, layered on established and new immigration streams from Ireland and other European countries (Dubuc 2012; Bijak et al. 2016). These largely labour-motivated migration streams were accompanied throughout our study period by subsequent family reunification from the Indian subcontinent in the 1980s, alongside new immigration flows from China, and African and European countries. Changes to the balance of European in- and out-migration and its diversity can be traced to several key moments between 1971 and 2011, including the UK joining the European Economic Community (EEC) in 1973, the transformation of the EEC into the European Union (EU) in the early 1990s, and EU enlargements in the 2000s, which saw a rapid increase in immigration to the UK, in particular from Poland and Romania (Dubuc 2012; Bijak et al. 2016; Blinder 2018). In the meantime, student immigration increased steadily during the latter stages of our study period, particularly from India and China, alongside flows from a diversity of African countries (Smith and Simpson 2015; Bijak et al. 2016). Most immigration to the UK has been for work, formal study and family reasons, with a comparatively small proportion migrating for asylum (ONS Digital 2015; Blinder 2018; Kone 2018).

Unsurprisingly, migration to the UK varies by region, with London persistently the most popular destination in our study period. That said, London’s dominance in the immigration stakes has been decreasing since the late 1990s, in favour of other regions (Kone 2018). Scotland’s 2011 resident population had a smaller share born outside the UK than its southern neighbour, yet the rate of increase was higher, with some two-thirds of the foreign-born population arriving since 2001 (Krausova and Vargas-Silva 2013; Smith and Simpson 2015). As with England and Wales, most immigration to Scotland is to urban areas, particularly Edinburgh, Glasgow and Aberdeen (Krausova and Varglas-Silva 2013; Smith and Simpson 2015). Settlement patterns also vary by country of origin. A distinction can be made between EU- and non-EU born migrants. While migrants from the EU tend to be more dispersed across parts of the country, including in agricultural areas, people with origins outside the EU more commonly settle in urban areas (ONS 2013; Kone 2018). Peach (2006) noted the interesting relationship between the micro-geographies of origin and destination for South Asians. Just as Indian, Pakistani and Bangladeshi migrants largely came from just a few districts in their respective countries, they likewise settled in specific neighbourhoods within metropolitan areas.

Of course, many of those residing in the UK who were born elsewhere have lived in the UK for some time—indeed, for most of their lives—and a considerable proportion have attained British citizenship. Immigration *created* Britain’s ethnic diversity, but its legacy of settlement and home-making, subsequent mobility within the country, new generations of UK-born ethnic minority groups, and inter-group mixing, continue to shape the geography of that diversity. In the final decade of our study (2001–2011), immigration continued to be the most significant mechanism of growth for the ethnic groups Other White, Black African, Chinese and Indian (Simpson and Jivraj 2015). While this period saw continued immigration of young adults in the White Irish ethnic group, the White Irish population declined, attributable to mortality of this ageing group, emigration, and changes in ethnic affiliation (towards the White British ethnic group) (Simpson and Jivraj 2015). For some ethnic groups with long histories in immigration, including Pakistani and Bangladeshi, and to a lesser degree Black Caribbean, between 2001 and 2011, population increases were mainly through more births than deaths (Simpson and Jivraj 2015).

While these demographic processes account for the growth and composition of ethnic diversity, the spatial patterns of this diversity are also (re)produced through internal migration within the country. The redistributive impact of within-UK migration over the last forty years has been significant (for an overview of major trends, see Champion 2016). Data advances since Robinson’s (1992) review of the paucity of studies of ethnic group mobility within the UK have facilitated research on internal migration by ethnicity during a period of growing policy-political interest in the residential location and (im)mobility of racialized minorities, inspired by concerns around integration and so-called ‘self-segregation’ (Finney et al. 2015). Such research has highlighted an unsurprising initial clustering of foreign-born populations in settlement areas, particularly in areas where people with common origins are already concentrated, but a subsequent spatial diffusion of ethnic minority groups (which include both the UK-born and non-UK born) over time (Peach, 1996; Simpson et al. 2008; Simpson and Finney 2009; Catney and Simpson 2010; Stillwell and McNulty 2012). This migration away from own-group clusters has acted to reduce residential segregation. Catney (2016b) showed how, between 2001 and 2011, there was a reduction in ethnic residential segregation for all ethnic minority groups, especially Black Caribbean, Indian, Mixed and Black African, and that neighbourhoods in urban areas like Leicester, Birmingham, Manchester and Bradford experienced a decrease in segregation for most ethnic groups. Johnston et al. (2016) pointed to decreasing segregation levels in London between 2001 and 2011 when observed at the Output Area level, yet more stable patterns at larger spatial scales.

Alongside decreases in residential segregation over time, ethnic diversity has increased, and has become more spatially complex. Rees and Butt (2004) identified two phases, whereby while between 1981 and 1991 ethnic minority groups were concentrating in metropolitan places, a decade later a process of deconcentration had begun, resulting in a “dramatic increase in ethnic diversity in all regions” (174). While ethnic diversity has grown in areas that have longer histories of diversity, such as London (Johnston et al. 2015), there has also been a spreading out of ethnic diversity between 1991 and 2011, with a growth of diversity outside of traditionally diverse spaces (Catney 2016a). The number of high-diversity—multi-ethnic—neighbourhoods in England grew considerably between 1991 and 2011 and, significantly, this diversity was *stable*, with over 95 percent of multi-ethnic neighbourhoods retaining their high-diversity state between 2001 and 2011 (Catney et al. 2020).

Considerably more attention has been paid to the ethnic geographies of England and Wales than of Scotland. Yet while Scotland has traditionally been home to lower levels of ethnic diversity, it has experienced a more rapid growth of several ethnic minority populations in recent years (Smith and Simpson 2015; Walsh et al. 2019). Similar to England and Wales, between 2001 and 2011 the growth of all ethnic minority groups was in areas where that ethnic group was the least clustered, with the exception of Chinese (attributed to student concentrations) (Smith and Simpson 2015). As with England and Wales, there is evidence that ethnic diversity has been spreading out to new locales, in a way that is “not creating polarised islands of specific groups, but rather a mosaic of differently mixed areas” (Smith and Simpson 2015: 103).

Despite this detailed bank of knowledge on the processes behind ethnic de-segregation, diversification, and the sub-national geography of diversity, spatially- *and* temporally-rich analyses have not yet been combined in a study that documents these changing micro-geographies of diversity. The analysis that follows uses finer-scale data than in previous studies, to explore the intricate tapestry of Britain’s ethnic geographies.

## The *PopChange* Resource

### Construction of Population Grids

The advantages of population grids and the lack of such data in Britain since 1981 (and indeed 1971, since no copies of the data can be found) provided motivation for a research project focused on the generation of consistent population grids from standard small area zones for each Census since 1971 (Lloyd et al. 2017). The project, *PopChange*, has provided 1 km grids for multiple demographic and socio-economic variables for all of Britain, including country of birth and ethnic group. The grids were generated using enumeration districts (1971–1991) or Output Areas (2001–2011 for England and Wales; 1991–2011 for Scotland)—the smallest available areas for each Census—with postcode data used to capture information on population densities within the source zones. More details on the method used to generate grids, as well as on population grids in general, are provided by Lloyd et al. (2017). In the UK, *PopChange* is the only resource that provides consistent small area data on ethnicity or country of origin for several time points, for the whole population. Grid data for Northern Ireland are not included in the study as, unlike the data for Britain (England, Scotland and Wales), they are not estimates and so they do provide a ‘like for like’ comparison. The mean population of all 239,855 1 km grid cells (populated and unpopulated) in the *PopChange* resource was 224 people in 1971; this has slowly but steadily risen each decade, to 256 people in 2011.

### Country of Birth and Ethnic Group Categorisations

While country of birth has been asked in the British Censuses (for both Scotland and England and Wales) since the start of our study period, it was not until 1991 that a question on ethnic group was included. It is apposite at this point to highlight the distinction between Census questions on country of birth—which ask if an individual has origins outside the UK (which might be recent or many decades ago)—and self-identified ethnic group, which may be influenced by, for example, one’s own or familial origins. The distinction between country of birth and ethnic group is important, and influences much of the interpretation of results in this paper. Country of birth should not be conflated with ‘immigrant’, since, as noted earlier, many people whose country of origin is outside the UK will have lived in the UK for most of their lives, and identify as British. Similarly, ethnic group should not be confused with country of birth; as an example, nearly half of the South Asian population living in Britain in 2001 were born in the UK (Peach 2006). It is not the purpose of this paper to define ethnicity, but we draw on two commonly-used variables that, broadly, capture ethnic identity and heritage (see, for example, Aspinall 2000; Phillips 2007; Finney and Simpson 2009; Mateos 2014; Jivraj and Simpson 2015). We use country of birth data to monitor trends between 1971 and 2011, and ethnic group data for 1991–2011.

Given inconsistencies in variable categories over time, some difficult decisions needed to be made in determining the population groupings to be included for analysis. Country of birth data were the most temporally-inconsistent, reflective of reconfigurations of nation states, and changes between Censuses in the ways that data on countries were collected and published. Ethnic group data were more easily harmonised, although these still required careful consideration given alterations in ethnic group measurement (questions, categories) *within* parts of Britain over time, and inconsistencies *between* parts of Britain at any time point. Table 1 shows the resultant set of (a) country of birth (hereafter CoB) categories for each time point between 1971 to 2011, and (b) ethnic groups between 1991 and 2011, with the number of people in each group in parenthesis. The final categorisations were a necessary compromise between comparability over time and granularity of detail. In the Censuses of 1971, 1981 and (to a lesser degree) 1991, many country origins are grouped in the published data into ‘Old Commonwealth’ or ‘New Commonwealth’, with limited or no capacity to consider countries within them. For CoB, only five fully comparable groups could be identified at all five Census time points (albeit with what became Bangladesh grouped in with Pakistan), plus an additional ‘Other’ category. This is extended to six groups for the period 1981–2011, with the inclusion of the recently-formed Bangladesh. In 1991, China appears as a separate origin, thus allowing comparisons between 1991 and 2011. For 2001 and 2011, there is a dramatically expanded set of common countries which could be used to undertake detailed geographical analyses. In this context, the focus is on long-term change and so the decision was taken to prioritise length of time over number of origins. This does present a significant compromise, especially given the importance of African and Caribbean countries (mostly absorbed into ‘New Commonwealth’ in the earlier Census periods) in the story of immigration to the UK.

**Table 1:** Population categories and sizes for Great Britain, for (a) country of birth, 1971–2011; (b) ethnic groups, 1991–2011. Numbers in bold refer to the population totals for each group, for England, Wales and Scotland combined

Country of birth group (a)	1971	1981	1991	2001	2011
UK	England, Scotland, Wales, Northern Ireland, UK part not specified, plus Isle of Man, Channel Islands (49,408,900)	England, Scotland, Wales, Rest of UK (50,171,931)	United Kingdom (including Channel Islands and Isle of Man) (50,931,607)	United Kingdom, Channel Islands and Isle of Man (52,274,535)	United Kingdom, Guernsey, Jersey, Channel Islands not otherwise specified, Isle of Man (53,520,678)
Ireland	Irish Republic (689,718)	Irish Republic (607,113)	Irish Republic (585,857)	Republic of Ireland (495,100)	Republic of Ireland (430,299)
Other Europe	Other Europe (excludes European countries within ‘New Commonwealth’) (611,688)	Other European Community, Other Europe (excludes European countries within ‘New Commonwealth’) (754,100)	Other European Community, Other Europe (excludes European countries within ‘New Commonwealth’) (736,577)	Europe minus United Kingdom and Republic of Ireland (1,043,642)	Europe minus United Kingdom and Republic of Ireland (2,462,617)
India	India (312,313)	India (391,702)	India (406,609)	India (466,537)	India (717,632)
Pakistan	Pakistan (Note: includes what became Bangladesh) (136,465)	Pakistan (188,204)	Pakistan (232,761)	Pakistan (320,939)	Pakistan (502,174)
*Bangladesh*	*Not available*	*Bangladesh (48,487)*	*Bangladesh (104,517)*	*Bangladesh (154,022)*	*Bangladesh (213,730)*
*China*	*Not available*	*Not available*	*China (23,289)*	*China (51,897)*	*China (167,834)*
Other	All other categories (1,317,365)	All other categories (inc. Bangladesh) (1,415,510)	All other categories (inc. Bangladesh and China) (1,733,418)	All other categories (inc. Bangladesh and China) (2,471,273)	All other categories (inc. Bangladesh and China) (3,735,382)

Ethnic group (b)	1991 England and Wales	1991 Scotland	2001 England and Wales	2001 Scotland	2011 England and Wales	2011 Scotland
White	White	White	White British, White Irish, Other White	White Scottish, Other White British, White Irish, Other White	White English/Welsh/ Scottish/Northern Irish/ British, White Irish, White Gypsy or Irish Traveller, Other White	White Scottish, Other White British (e.g., Welsh), White Irish, Polish, White Gypsy or Irish Traveller, Other White
	(**51,641,478**)		(**52,478,455**)		(**53,291,294**)	
Indian	Indian	Indian	Indian	Indian	Indian	Indian
	(**835,873**)		(**1,051,659**)		(**1,445,660**)	
Pakistani	Pakistani	Pakistani	Pakistani	Pakistani	Pakistani	Pakistani
	(**475,019**)		(**746,532**)		(**1,173,890**)	
Bangladeshi	Bangladeshi	Bangladeshi	Bangladeshi	Bangladeshi	Bangladeshi	Bangladeshi
	(**162,205**)		(**282,892**)		(**450,988**)	
Black Caribbean	Black Caribbean	Black Caribbean	Black Caribbean	Black Caribbean	Black Caribbean	Caribbean or Black
	(**496,297**)		(**565,825**)		(**601,362**)	
Black African	Black African	Black African	Black African	Black African	Black African	African
	(**206,899**)		(**484,682**)		(**1,019,263**)	
Chinese	Chinese	Chinese	Chinese	Chinese	Chinese	Chinese
	(**153,318**)		(**243,314**)		(**426,843**)	
Other	Other Asian, Other Black, Other	Other Asian, Other Black, Other	Other Asian, Other Black, Other, White and Black Caribbean, White and Black African, White and Asian, Other Mixed	Other South Asian, Other Black, Other, Any Mixed Background	Other Asian, Other Black, Other, White and Black Caribbean, White and Black African, White and Asian, Other Mixed, Arab	Other Asian, Other, Mixed or Multiple ethnic groups, Arab
	(**657,048**)		(**1,247,390**)		(**2,959,468**)	

(a) Rows in italics are for alternative population counts used in selected analyses (identified within the main text). Pakistan includes Bangladeshi in 1971. Other refers to the 1971 base; it includes Bangladeshi in 1981–2011 and China in all Census years. Descriptions vary between Census years and the labels follows those used by the Census offices

*Sources*: (a) Censuses of England and Wales and Scotland 1971 (Table SAS08), 1981 (Table SAS04), 1991 (Table SAS07), 2001 (Table UV08), 2011 (Table QS203); (b) Censuses of England and Wales and Scotland 1991 (Table SAS06), 2001 (Table KS006), 2011 (Table KS201)

The ethnic group question saw significant modifications between 1991 and 2001, with some additional adaptions in 2011 (Table 1). The 1991 Census included the fewest response categories, and was consistent across Britain. In 2001, the White ethnic category was broadened to include White Irish and Other White, and Mixed ethnic categories were included. Scottish respondents could, for the first time, tick a ‘White Scottish’ box. By 2011, the White British category in England and Wales was disaggregated into White English/Welsh/Scottish/Northern Irish/British. White Gypsy or Irish Traveller and Arab constituted new categories in England and Wales, as well as White Polish in Scotland. There are some additional differences in ethnic group categorisation between England and Wales and Scotland in 2011—the Black African category in England and Wales corresponds most closely to African in Scotland, while Black Caribbean in England and Wales most closely matches Caribbean or Black in Scotland.

## Forty Years of Change in Ethnic Diversity

**Fig. 1:**
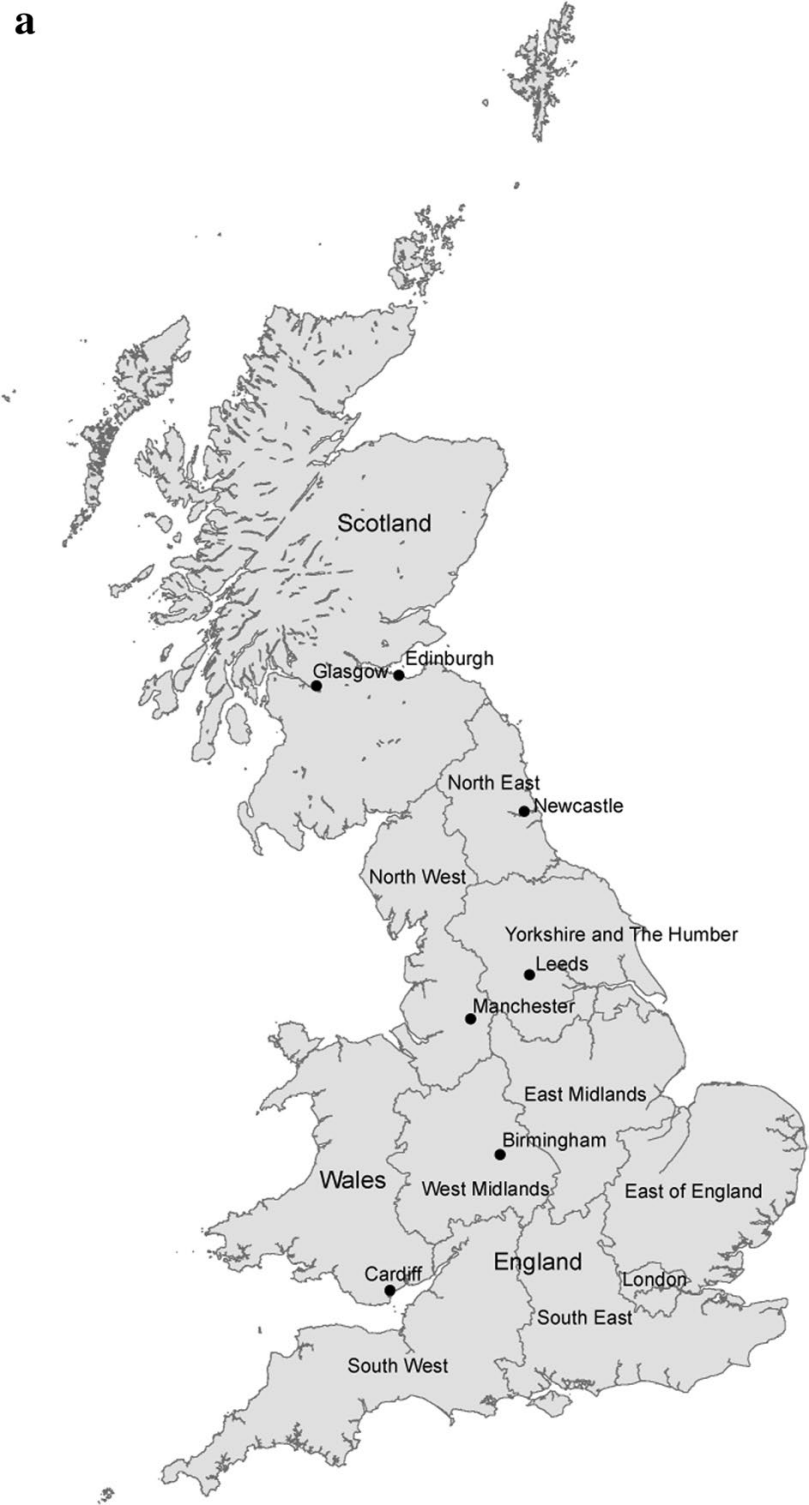

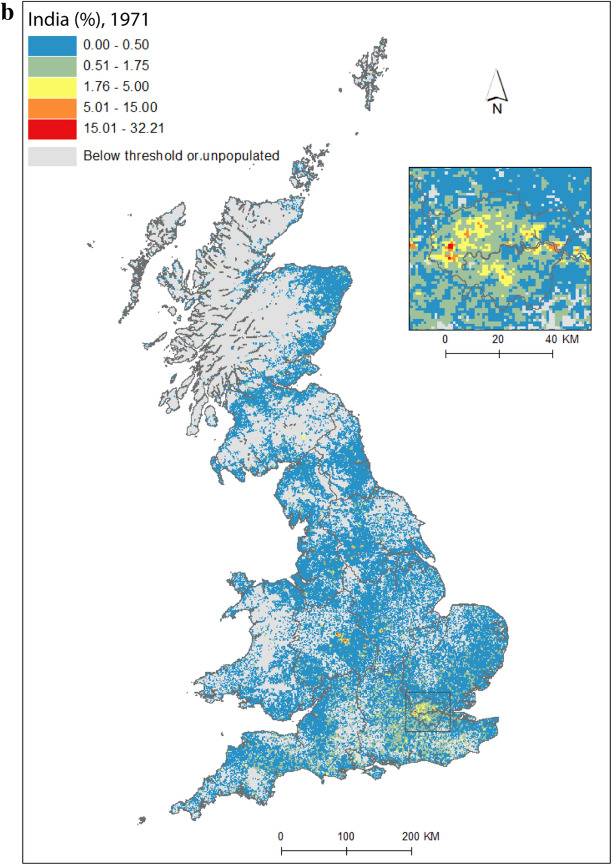

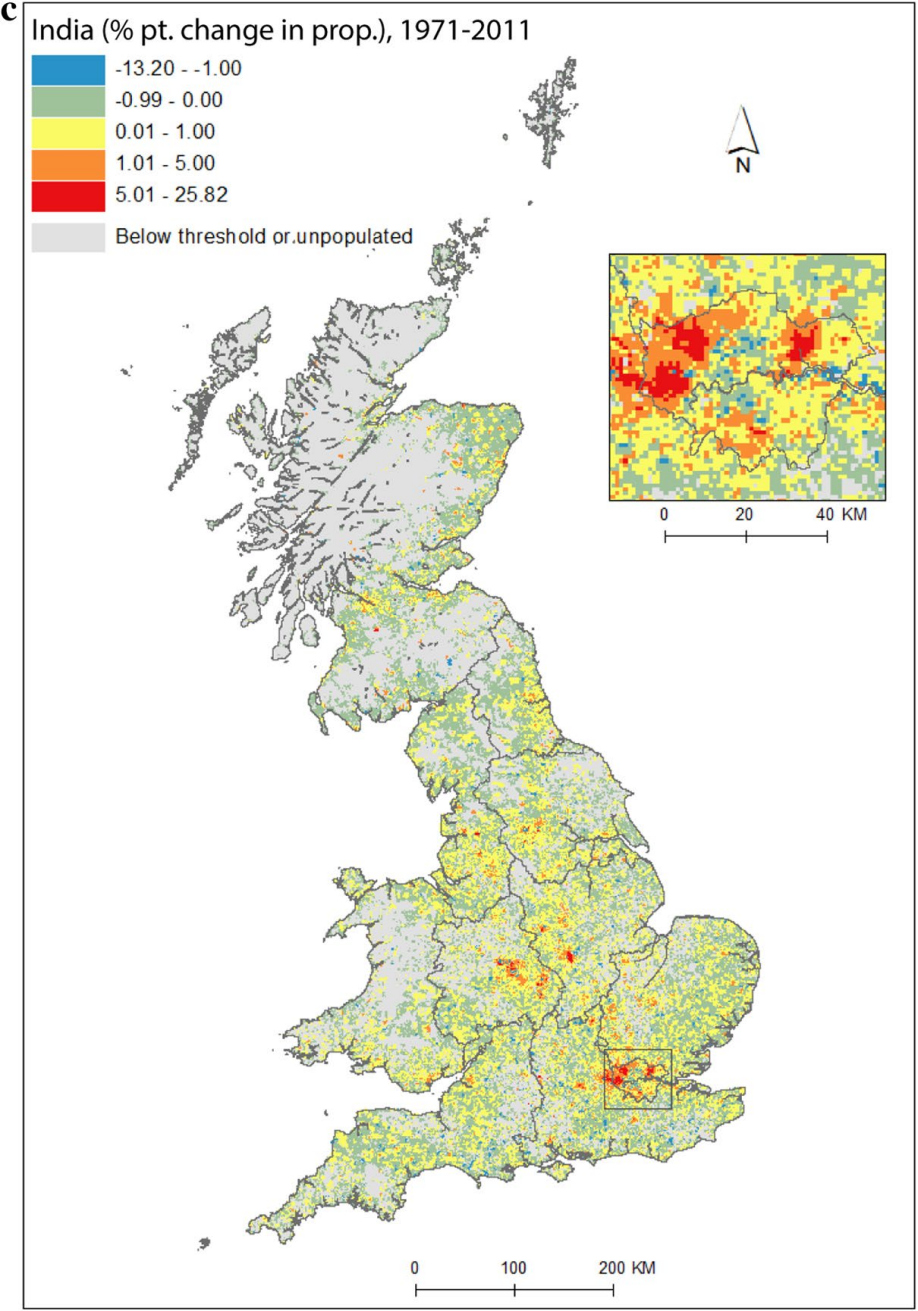

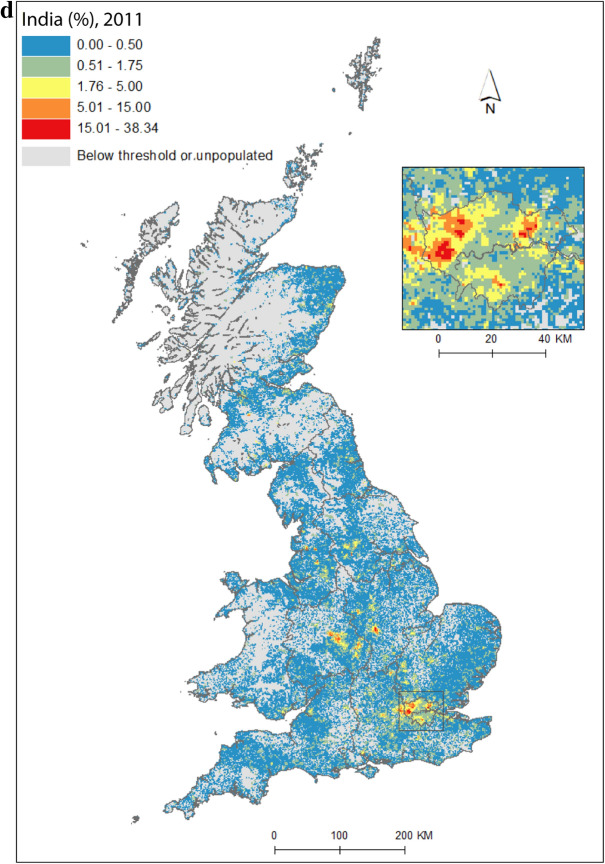
**a** regions and selected cities; **b** percentage of 1971 residents in Britain born in India; **c** percentage point change in the proportion born in India, 1971–2011; **d** percentage of 2011 residents in Britain born in India. *Sources* Censuses of England and Wales and Scotland 1971 (Table SAS08), 2011 (Table QS203). (Color figure online)

As a pathway into our analysis, we first present an application of the data by concentrating on India, the single country of origin group^3^ that has had the largest, or amongst the largest, population across all *PopChange* time periods. Figure 1a shows regions and selected places, for reference. Figure 1(b) is the proportion of people (of the total population) who were born in India and living in Great Britain at the start of our study period, Fig. 1c shows percentage point change in the proportion of people born in India between 1971 and 2011, and Fig. 1d illustrates the residential geography these changes had shaped by the end of the study period. The maps clearly demonstrate the intricate spatial detail that gridded data unlock. Indian settlement in the UK has traditionally been concentrated in London, the midlands, and the north-west of England (Peach 2006). These broad regional patterns can be seen in Fig. 1, but the *micro*-geographies of growth are also clearly discernable, and reveal much more than data at a lower spatial resolution. While labour- and family-related migration drew Indian migrants to the UK, it was very particular *parts* of cities, neighbourhoods and streets that were transformed by these migrations. Subsequent immigration and family-building will have resulted in growth in the settlement areas identified for 1971 (Fig. 1b), but we also observe new pockets of Indian communities, in particular across mid- and northern England (Figs. 1c, d), likely given socio-spatial mobility away from these settlement areas (Catney and Simpson 2010) and new immigration flows, for example by students.

Figure 1 reveals the changing geographies of settlement and dispersal for people from one country of origin. We next consider how the stories from *several* countries of origin interweaved to shape Britain’s geography of *diversity*. Diversity is measured using the reciprocal diversity index (RDI; see Catney 2016a for an application to ethnic group data). The RDI for grid cell $i$ is defined as:

$$RDI_{i} = \left( {\left( {1/\mathop \sum \limits_{m = 1}^{M} \left( {\frac{{N_{im} }}{{N_{i} }}} \right)^{2} } \right) - 1} \right)/(M - 1)$$

where $m$ is a population group; $M$ is the number of population groups; $N_{im}$ is the number of persons in group $m$ in grid cell $i$. Subtraction of one and division by *M*-1 constrains the index to the range zero to one. A large RDI value in a given grid square indicates higher levels of diversity—that is, a spread of members of multiple countries of origin, or multiple ethnic groups. A value of one would indicate an even spread of groups—in the case of five groups, this means that each group comprises 20 percent of the total. A relatively high RDI value can thus be taken as an indicator of greater inter-group mixing since it represents the presence of members of several groups. Grid cells can have dramatic variations in population density, and this means that proportions computed from grid cell counts may be unreliable in some areas where the population density is very low. The present study is based on the inclusion of all populated grid cells (that is, those estimated to contain at least one person). In practice, this means that all cells with more than 0.5 people are used in the calculations. Experimentation with different threshold values (for example 25 people) shows that the same general trends emerge irrespective of the population cut-off used, and that the results of our analyses are robust to changes in thresholds.

The median CoB RDI values increased between all time points (1971 = 0.085; 1981 = 0.094; 1991 = 0.101; 2001 = 0.114; 2011 = 0.136), as did the median ethnic group RDI values (1991 = 0.032; 2001 = 0.047; 2011 = 0.065), representing a Britain-wide steady increase in diversity over time. The numbers of groups used to compute RDI in the CoB and ethnic groups differ, so comparisons of the two sets of figures should be undertaken with caution. It is, however, interesting to note that the median RDI values for CoB are higher than for ethnic group. One possible reason for this difference is the inclusion of multiple European-origin groups within the White ethnic group, but a separate Other Europe category for CoB—in effect, this component of diversity is (in relative terms) suppressed in the ethnic group analysis.

**Fig. 2:**
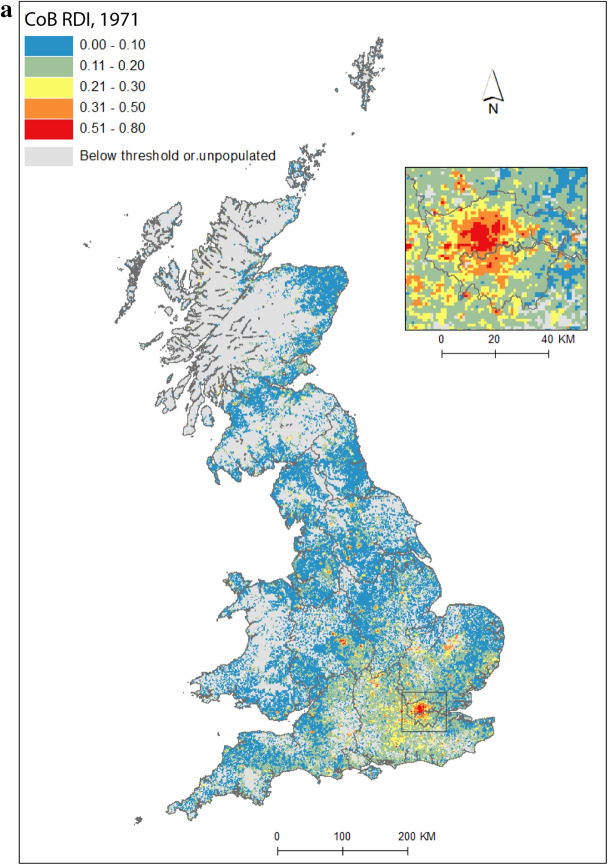

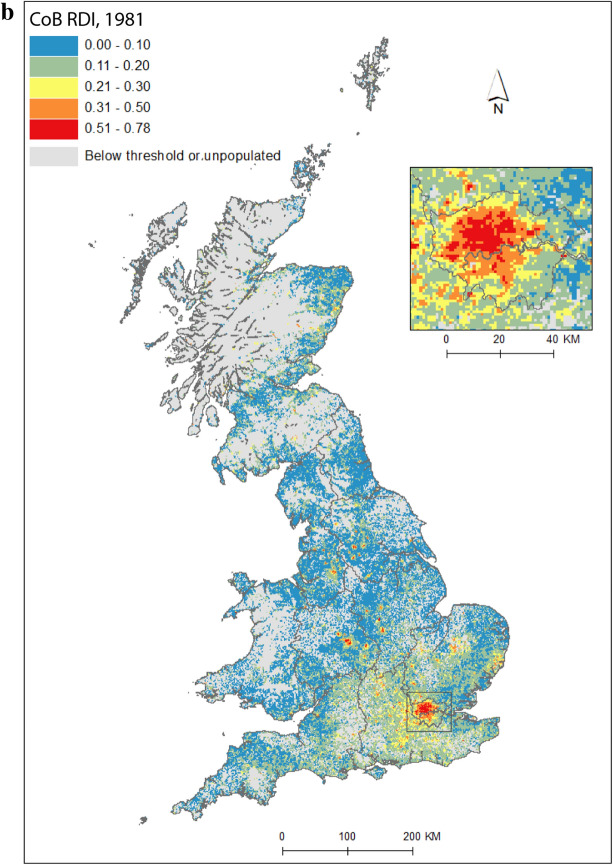

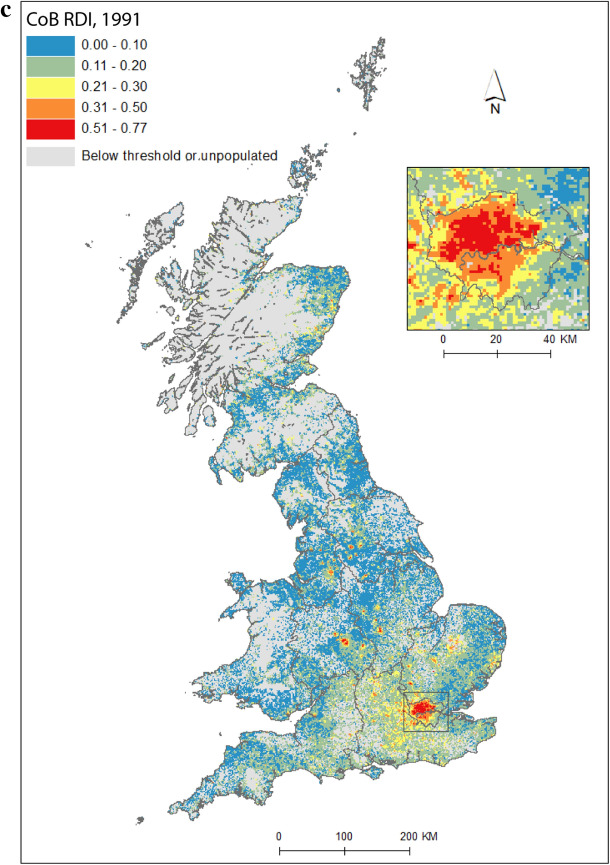

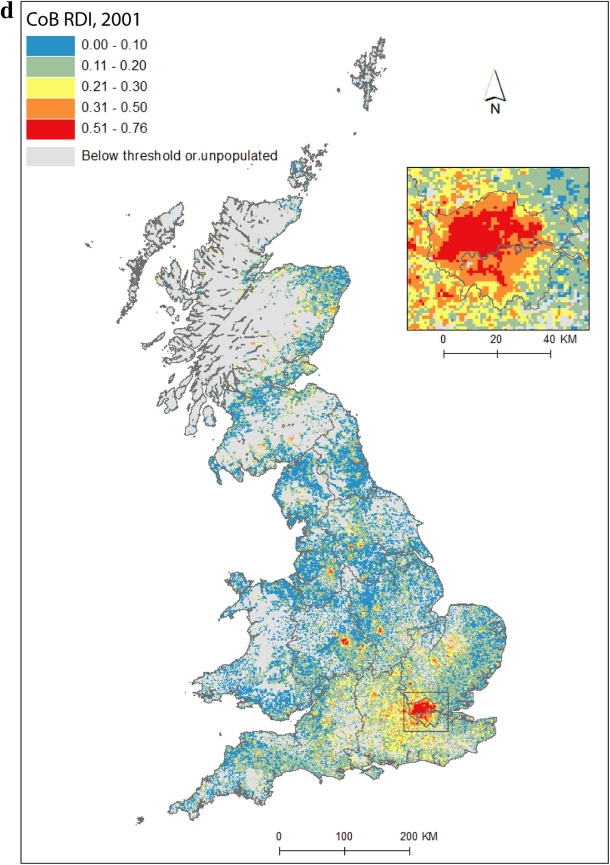

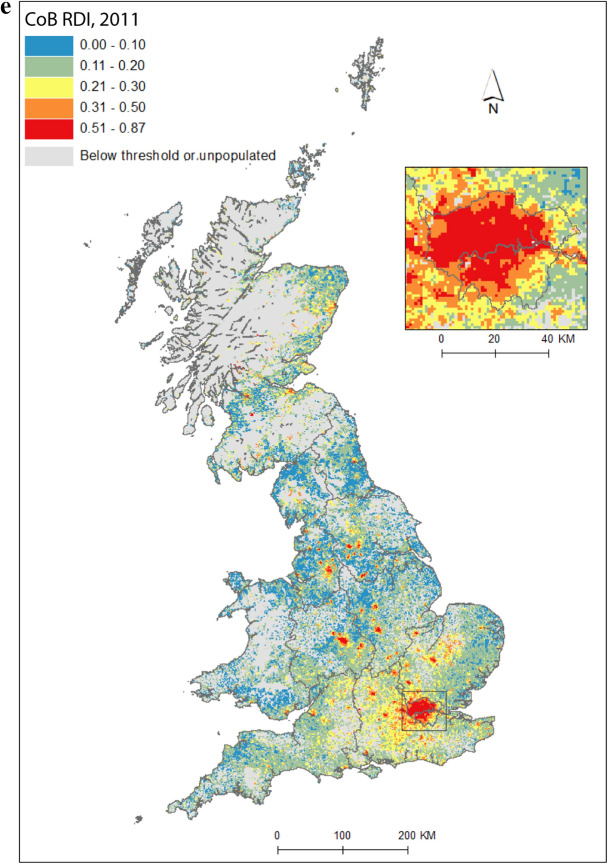

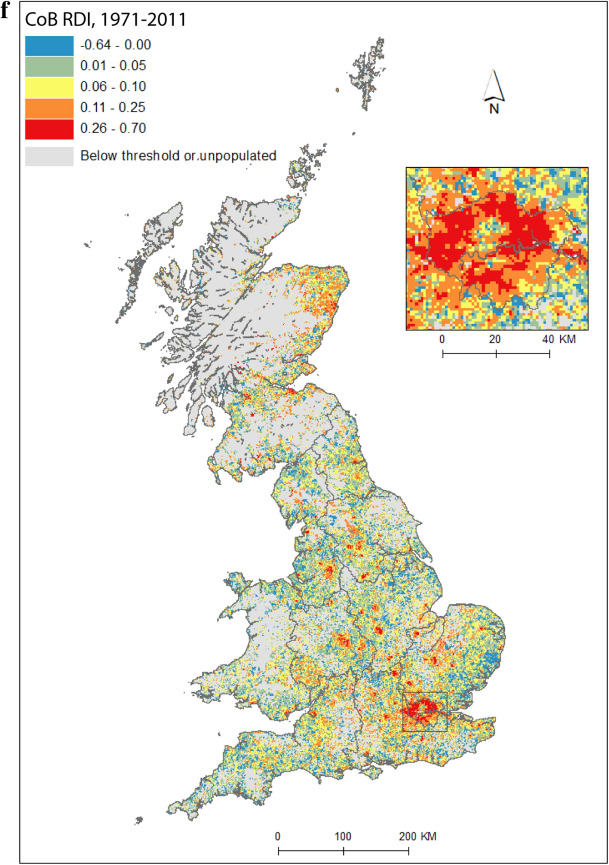
Reciprocal Diversity Index (RDI) for country of birth categories, **a** 1971; **b** 1981; **c** 1991; **d** 2001; **e** 2011; and **f** change in RDI values, 1971–2011. *Sources* Censuses of England and Wales and Scotland 1971 (Table SAS08), 1981 (Table SAS04), 1991 (Table SAS07), 2001 (Table UV08), 2011 (Table QS203). (Color figure online)

Figure 2 maps diversity using the CoB groupings UK, Ireland, Other Europe, India, Pakistan (for 1971 this includes what became Bangladesh), and Other (see Table 1 for description of the groupings). Maps are included for each of the five time points in the study period, as well as change between 1971 and 2011. Figure 2(a) shows the landscape of diversity for 1971 which, at this point in time, had been mainly shaped by immigrant settlement and some familial growth (see Sect. 3). London, a hub for new arrivals, shows the greatest diversity, and Birmingham exhibits more modest yet discernable diversity. Smaller pockets of diversity in the north of England begin to emerge by 1981 (Fig. 2b), and become more easily identifiable by 1991 (Fig. 2c). By 2001 (Fig. 2d), we find a geography of diversity that is the outcome of population growth and internal migration, but the next ten years were to see significant further increases in diversity across Great Britain (Fig. 2e).

Figure 2(e) provides a lens into the most diverse small areas of all the maps. Concentrating on the category of highest category of ethnic diversity (>0.5), we see a distinct bias towards towns and cities, with London home to by far the most diverse neighbourhoods. By 2011, many parts of the capital had relatively high proportions of residents with origins outside the UK. This diversity spills over London’s borders; high or mid-ranging levels of diversity can be found throughout much of its hinterland, and indeed throughout the south-east of England. Birmingham is clearly home to the next largest cluster of diverse small areas. While the most diverse grid squares are exclusively metropolitan, moderate levels of diversity can be found in less urban locales. The distinctive spatial pattern of higher levels of urban diversity alongside a ring of slightly lower diversity in the suburbs is discernable throughout many of England’s cities—particularly in London, Birmingham, Manchester, Leicester, Nottingham and Bradford. Each of these locales have a different history of diversity. London has been shaped by a long history of immigration from an immense diversity of origins (Johnston et al. 2015). Diversity in Leicester grew from Caribbean and Indian migration for post-war industrial labour opportunities, East African Asian immigration in the 1970s, more recent Eastern European immigration, and Asian student immigration (Leicester City Council 2019).

Military bases in and around the south, midlands and south-east of England explain other patches of diversity shown in Fig. 2e. In this same map, diversity in Scotland and Wales in 2011 is clearly still considerably more modest than in England. Some of the new diversity emerging in these parts of Britain will be masked by employing the same range of diversity categories as for their much more diverse English neighbour. For Wales, Cardiff city, alongside some university towns and cities, is most diverse. Scotland’s major cities of Edinburgh and Glasgow, as well as Aberdeen and the university town of St Andrews, are more diverse than other locales. Scotland is temporary home to a larger proportion of international students than England and Wales, with the result that many of Scotland’s most diverse neighbourhoods can be found in university towns and cities (see also Smith and Simpson 2015; Walsh et al. 2019). There are many areas of (especially) rural England (notably in the south-west, midlands and north-east), rural southern Scotland, and much of Wales, which have low levels of diversity—although there is growth in diversity even in parts of these regions.

Figure 2(f) shows how diversity in the period 1971–2011 is characterised not only by growth, but also by an increasing spatial intricacy. Large areas of Great Britain experienced an increase in diversity, and new spaces of diversity emerged, resulting in a more complex landscape of mixedness. More grid cells—neighbourhoods—are diverse than ever before (note in particular the increase in grid cells that have RDI values in the highest range of values in Fig. 2, of 0.51 or above). This mixing has reached new corners of Britain, including outside major urban areas. The map also shows how *decreases* in diversity are rare, and tend to be confined to rural locales, particularly coastal areas in Wales and in the south-east and north-west of England. Most of Scotland’s small areas have experienced a growth in diversity, albeit from a lower base than for England. Edinburgh was more diverse than Glasgow in 2001 and 2011, but the rate of growth of diversity in Glasgow was greater over the period.

Figure 2 captures the diversity of countries of origin. This includes recent immigrants, and people who made Britain their home many decades ago, including before our study period began. The mechanisms that shape the changing geography of diversity shown are therefore a combination of new immigration, emigration, migration within the country, and the growth or decline of groups through the balance of fertility and mortality. Of the 1740 grid cells which had an RDI value of greater than 0.5 in 2011, 65 percent changed most between 2001 and 2011, 19 percent between 1971 and 81, 10 percent between 1991 and 2001, and six percent between 1981 and 1991. The most impactful changes to Britain’s diversity thus occurred in the final and, to a much less degree, earliest periods of the study. Between 1971 and 1981, labour and family migration added to the diversifying British population, with a faster rate of change than in later periods when our measure of diversity will have grown from an already more diverse base. The most dynamic period of change, 2001 to 2011, has been shaped by continued immigration, including via an increasingly international Higher Education sector, and the dispersal of long-established families into new, previously less diverse, suburban and rural locales (Catney 2016a).

**Fig. 3:**
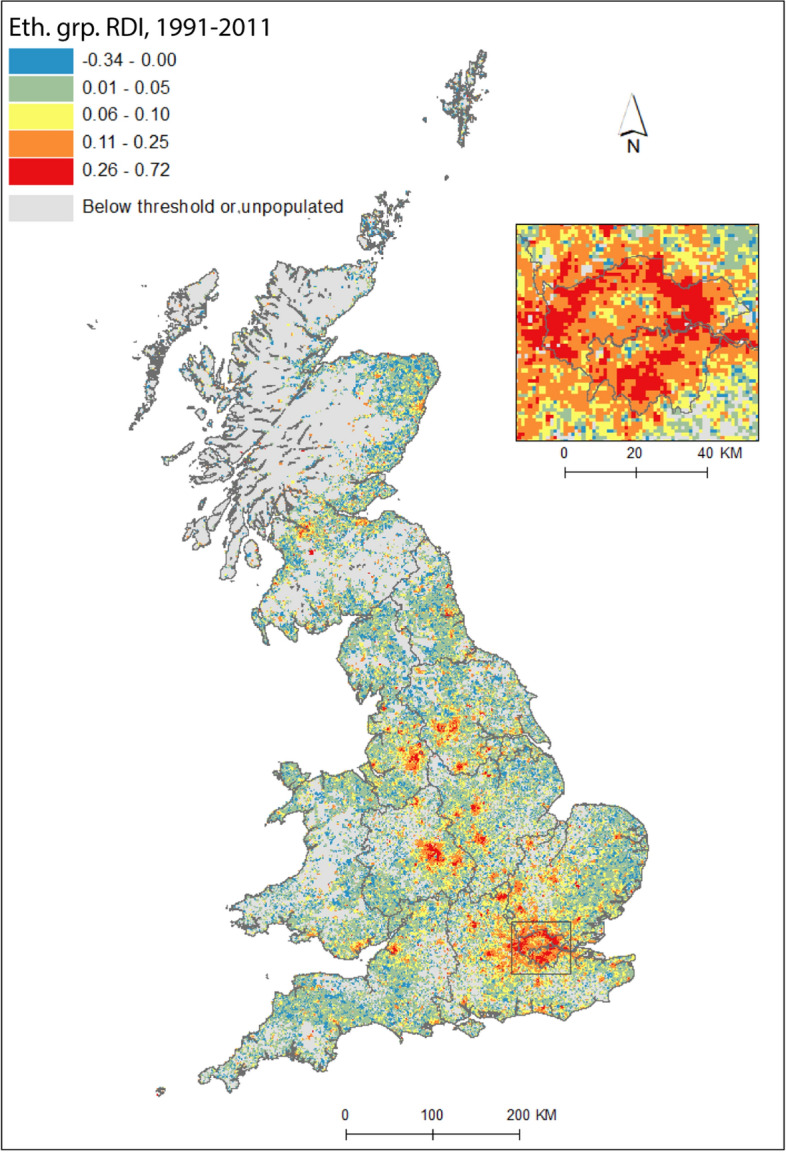
Change in Reciprocal Diversity Index for ethnic group, 1991–2011. *Sources*: Censuses of England and Wales and Scotland 1991 (Table SAS06), 2011 (Table KS201). (Color figure online)

Figure 3 reveals the landscape of diversity not exclusively of people born outside the UK, but also those who are UK-born and may have a heritage—for example, a parent’s, or grandparent’s birthplace—in another country. For the sake of space, we only map *change* in ethnic group diversity between 1971 and 2011. The patterns of change in the geographies of ethnic group diversity are in many ways similar to that of CoB (Fig. 2f); we see an urban dominance, but also growth outside traditionally diverse spaces, in suburban and rural locales. As with CoB, low levels of growth of, and decreases in, diversity by ethnic group are also largely the reserve of rural and coastal areas. However, there are also some notable distinctions from CoB in the spatial patterns of changes in ethnic group diversity. Growth has been even more pronounced in the major urban centres outside of London, at this very fine spatial scale. We observe increases in ethnic diversity in more grid squares in Birmingham, Leicester, Manchester, Bradford, Glasgow and Edinburgh, than for CoB. The spatial extent of this growth is also greater, incorporating more of the hinterlands of towns and cities outside London. In contrast, London has a slightly more modest picture of increasing diversity as measured by ethnic group than for CoB. London is a dynamic city which continues to attract the lion’s share of immigration to the UK (Kone 2018), its attraction partly inspired by its diversity. The growth of diversity of London’s hinterland, on the other hand, as with other urban areas, might be driven mainly by the dispersal outside of urban centres of UK-born ethnic minorities, towards traditionally less diverse locales (see Simpson and Finney 2009). Catney et al. (2020) demonstrated how England's multi-ethnic neighbourhoods—spaces where no one ethnic group were in a majority and at least five ethnic groups had representation—were most common in London, but also grew outside the captial between 1991 and 2011, in, for example, Birmingham and Greater Manchester.

**Fig. 4:**
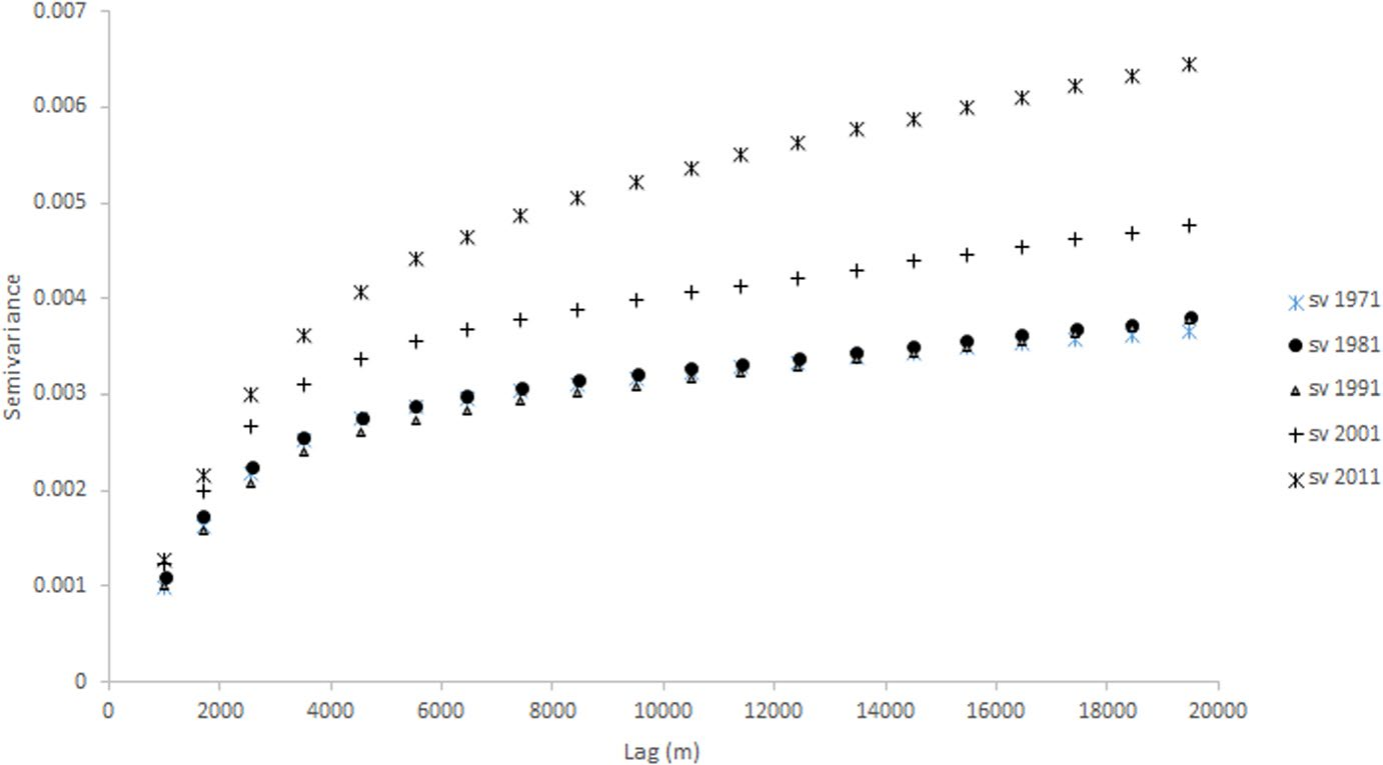
Variograms estimated from Reciprocal Diversity Index values for country of birth for 1971, 1981, 1991, 2001, and 2011. *Sources*: Censuses of England and Wales and Scotland 1971 (Table SAS08), 1981 (Table SAS04), 1991 (Table SAS07), 2001 (Table UV08), 2011 (Table QS203)

The RDI maps reveal a ‘spreading out’ of diversity from urban areas with relatively high levels of diversity at all time points, and growth in some suburban and rural locales. The trends observed reflect the changing spatial scales of diversity, which, with important exceptions (e.g., Wright et al. 2014; Ellis et al. 2018) has received considerably less attention than have the scales of residential segregation (see, for example Reardon et al. 2009; Fowler 2016; Johnston et al. 2016). These scales can be quantified using one of a number of ‘structure functions’, including the variogram (see Webster and Oliver 2007). Variograms are used widely in modelling physical properties, but only rarely in analyses of populations (Lloyd 2015); their use in exploring ethnic diversity is novel. The variogram relates the distance (or lag) between paired observations (in this case, 1 km grid cells) to half the average squared difference (the semivariance) between the values attached to them (here, RDI values). Variograms of most population characteristics tend to have small semivariance values at short distances and larger semivariance values at larger distances (see Lloyd 2015 for examples). Variograms were estimated for each Census year for the CoB RDI values (Fig. 4). These provide a graphical summary of the spatial scale or extent of diversity. Changes in the geographical scale of diversity can be measured, providing a powerful means to show over what periods of time changes were largest.

There are two key features to note on the variograms—the magnitude of the values (the semivariances themselves) and the lag distances at which there are, effectively, breaks of slope. In the case of the variogram for 1981, for example, there is a clear change in semivariance values at a lag distance of around 4 km. These breaks of slope are termed ranges in non-linear models fitted to variograms, and they indicate major spatial scales of variation corresponding to ‘clumps’ of similar values. The variograms for 1971, 1981, and 1991 have very similar forms. In contrast, the variogram for 2001 has a larger maximum value, and that for 2011 has a larger value still. The ‘range’ values also increase between 1991 and 2001, and between 2001 and 2011. This shows that the spatial scale of diversity was relatively consistent between 1971 and 1991, but it increased between 1991 and 2001, with the largest increase between 2001 and 2011. The increasing maximum semivariance values indicate an increase in the differences between highly diverse urban areas and elsewhere, with diversity moving from urban cores to reach suburban areas.

This quantification of scale differences, not easily observed in cartographic form, suggests an increasing momentum in the growth of diversity, with a clear increase between 1991 and 2001, and even greater growth between 2001 and 2011. Variograms offer an alternative perspective to existing measures based on computing indices for a set of different geographical bandwidths (neighbourhoods of different size), applied in the main to studies of segregation, rather than diversity (see, for example, Reardon et al. 2008). With variograms, specific scales of variation (ranges) can be identified, and these are arguably more effective summaries than ratios between measures for two discrete bandwidths.

Population grids offer particular advantages in the exploration of spatial scale—since the units (grid cells) are of equal size it is straightforward to analyse changes by spatial scale. This can be done either by (for example) computing variograms with regular lag sizes (0–1 km, > 1–2 km,…), or by aggregating cells into progressively larger cells and examining the proportions of variance accounted for at each level of aggregation. Variogram analysis also provides a means to assess just how small zones would need to be to allow for a meaningful analysis of populations. In the examples presented here, it is clear that there is a large amount of spatial variation at distances smaller than 2–3 km—there are large differences in the semivariance values at 1, 2, and 3 km. This suggests that using zones with (average) widths larger than this would result in a considerable loss of information. A more formal approach to identifying appropriate zone sizes is offered by variogram deconvolution (see Goovaerts 2008).

## Micro-Scale Changes in Residential Segregation

In this section, the focus is on residential segregation—how far members of different groups (by country of origin or ethnicity) share residential neighbourhoods, and how spatial integration has changed over time. Here, segregation is measured using the Index of Dissimilarity (*D*; Massey and Denton 1988). *D* takes values from zero (all grid squares comprise members of one group *or* the other) and one (all grid squares have the same proportions of each group; for example, 55 percent of group *m* and 45 percent group *n*). *D* is defined as:

$$D = 0.5 \times \mathop \sum \limits_{i = 1}^{{N_{I} }} \left| {\frac{{N_{im} }}{{N_{m} }} - \frac{{N_{in} }}{{N_{n} }}} \right|$$

where $m$ is population group $m$; $n$ is population group $n$; $i$ is a zone (grid square); $N_{I}$ is the number of zones; $N_{im}$ is the number of persons in group $m$ in zone $i$; $N_{m}$ is the number of persons in group $m$. *D* is calculated for each CoB or ethnic group compared to the rest of the population. Table 2 shows Index of Dissimilarity (*D*) values for 1971, 1981, 1991, 2001 and 2011 for each of the major CoBs used in the study, with additional rows for Bangladesh and China (as explained in Sect. 4.2; see also Table 1). Table 3 shows the same measure of segregation for ethnic group, from just 1991 onwards, given data availability.

**Table 2:** Index of Dissimilarity, country of birth, 1971–2011

Country of Birth	1971	1981	1991	2001	2011
UK	0.417	0.423	0.432	0.444	0.453
Ireland	0.389	0.383	0.392	0.349	0.317
Other Europe	0.365	0.345	0.333	0.350	0.380
India	0.557	0.569	0.564	0.555	0.536
Pakistan	0.724	0.733	0.725	0.703	0.684
Other 71 base	0.453	0.454	0.460	0.480	0.460
Bangladesh	n/a	0.774	0.761	0.738	0.682
China	n/a	n/a	0.572	0.578	0.539
Other 91 base	n/a	n/a	0.450	0.471	0.449

Notes: Pakistan includes Bangladeshi in 1971. Other 71 base includes Bangladesh in 1981–2011 and China in all Census years. Other 91 base excludes Bangladesh and China in 1991–2011. *D* is for each group versus all the rest

Sources: Censuses of England and Wales and Scotland 1971 (Table SAS08), 1981 (Table SAS04), 1991 (Table SAS07), 2001 (Table UV08), 2011 (Table QS203)

**Table 3:** Index of Dissimilarity, ethnic group, 1991–2011

Ethnic group	1991	2001	2011
White	0.628	0.598	0.571
Indian	0.670	0.636	0.582
Pakistani	0.758	0.720	0.702
Bangladeshi	0.765	0.736	0.687
Black Caribbean	0.712	0.690	0.639
Black African	0.727	0.719	0.610
Chinese	0.463	0.458	0.450
Other	0.504	0.447	0.457

Notes: *D* is for each group versus all the rest

Sources: Censuses of England and Wales and Scotland 1991 (Table SAS06), 2001 (Table KS006), 2011 (Table KS201)

The most uneven residential geography by CoB (Table 2) is for people born in Bangladesh, with segregation values ranging from 0.682 (in 2011) to 0.774 (1981). The Pakistan category includes Bangladesh in 1971, and this group had the highest segregation value for that year (0.724). Given the longer history of origins in immigration for the Pakistani and Bangladeshi populations, a commonality in residential patterns with those who identify as Pakistani or Bangladeshi, regardless of place of birth, might be expected. This is confirmed in Table 3; segregation levels are highest for the Pakistani and Bangladeshi ethnic groups. The residential geographies of the Pakistani and Bangladeshi groups have traditionally been spatially concentrated. Peach (1996) noted how, in 1991, nearly one-quarter of the Bangladeshi ethnic group lived in the London Borough of Tower Hamlets, a settlement pattern that can be found some twenty years later (Catney 2017). Of course, many of the people in the Pakistani and Bangladeshi ethnic groups will also have been born in either Pakistan or Bangladesh, but aside from these common populations, there are several other reasons why a temporal consistency in spatial patterns might be expected. Higher rates of family-linked migration (ONS 2013) will maintain long-established settlement geographies. Indeed, 1981 represents a peak in segregation levels for the South Asian groups (Table 2), perhaps reflecting an initial period of clustering following arrival in the UK in the few decades previous. Peach (1996) highlighted the importance of familial and social networks, alongside other factors such as religion and diet, in explaining higher levels of spatial concentration among the Pakistani and Bangladeshi populations. These mechanisms operate alongside discrimination into certain housing types and locales, and persistent poverty—inequalities of opportunity that have not improved in the following decades (see the volume by Jivraj and Simpson 2015). Young adults in these ethnic groups also have relatively low levels of residential mobility, related to preferences in partnership and family formation and for remaining in the family home while in further or higher education (Finney 2011).

Yet there was a noticeable *decline* in residential segregation levels for the Pakistani and Bangladeshi ethnic groups, as well as for Pakistan and Bangladesh countries of birth. In particular, there was a relatively sharp reduction in segregation between 2001 and 2011 for Pakistan CoB, and for Bangladesh CoB and the Bangladeshi ethnic group. Increasing time since early periods of immigration and settlement, decreasing fertility rates (Dubuc 2012), and socio-spatial mobility away from urban centres (Simpson and Finney 2009; Catney and Simpson 2010), will have contributed to the observed reduction in segregation for these groups. Indian segregation is lower than the other South Asian groups, and decreasing, particularly as measured by ethnic group (Tables 2, 3), which includes a considerable proportion of people born in the UK. These decreases might be attributable to the group’s relative socio-economic advantage in the labour and housing markets compared to some other ethnic groups (Jivraj and Simpson 2015). Student migration from India to UK university towns and cities, especially in the latter years of the study period (ONS 2013; Blinder 2018), will play an important role in maintaining some residential clusters in particular parts of the country.

Ireland and Other Europe are the CoB groups that are consistently the least spatially concentrated. This is likely explained by their more rapid socio-spatial integration and, in the case of Ireland, long history of immigration to the UK, whereby many people will have settled in the UK at start of our study period, if not earlier. The heterogeneous Other Europe category masks considerable variation in motivations for immigration, periods of arrival, settlement patterns, and housing and labour market experiences within the UK, and includes Eastern European ‘accession’ migrants moving to the UK mainly for work and, for example, German-born migrants, many of whom are the children of UK service personnel stationed in Germany. The former group has tended to follow a relatively spatially dispersed pattern of settlement, towards agricultural areas, while the latter has a more variable distribution—in London and other metropolitan places, as well as military bases (ONS 2013). Chinese segregation is amongst the lowest, and stably so over the study period, for both CoB and ethnic group, a finding consistent with other studies for more recent time periods (e.g., Catney 2016b, 2017). The Black African and Caribbean groups are not identifiable in the CoB data (see Sect. 4.2), but show, for ethnic group, steadily decreasing residential segregation levels at this spatial scale.

People born in the UK are increasingly exposed to people born elsewhere; it therefore seems a paradox that as the country has become more diverse, the UK-born appear *more* segregated (Table 2). Given that *D* is computed for each group compared to the *rest* of the population, the *D* figures for the UK-born thus also reflect changes in the non-UK born population. In simple terms, the UK-born population was fairly evenly distributed in 1971, with most non-UK born people living in particular urban locales. As diversity spreads—and more people born outside of the UK reside in locales outside of metropolitan cores (including increasingly in the suburbs of larger urban areas; Figs. 2 and 3)—the non UK-born population, taken *collectively*, and thus the UK-born population, is increasingly unevenly distributed. Of course, there is no such ‘collective’ population, and, in practice, as *individual* non-UK populations become more geographically dispersed, their initially clustered (and urban-focused) population distributions become more geographically even. While it is not strictly fair to compare across Tables 2 and 3, it is interesting to note how segregation levels for the two majority populations differ; the UK CoB group (Table 2) has considerably lower segregation than the White ethnic group (Table 3). The former, of course, includes ethnic minority groups, as well as the White British population; by this measure, the UK-born White and ethnic minority populations appear to be more residentially integrated than the White ethnic group.

## Summary and conclusions

For this special issue on “Exploiting Fine-scale Data in Modeling Migrants’ Settlement Patterns in Europe” we set out to explore the potential of population grids for analysing long-term change in ethnic/racial geographies. Unlike using standard administrative zones for analyses of change over time, population grids are not based on a population structure at one time point (or adapted for past or future populations). Additionally, they offer a ‘natural’ way of exploring population change since they show which areas are truly neighbours, in contrast to standard zones which may share boundaries but, in reality, be separated by unoccupied areas, such as agricultural land. By showing unpopulated areas, grids more accurately depict population distributions, and population growth. Yet population grids are often overlooked in studies of long-term trends in ethnic diversity and segregation.

We demonstrate the utility of population grids using a novel (and freely-available) resource, *PopChange*, which provides spatially fine-grained gridded data on country of birth for 1971–2011 and ethnic group for 1991–2011 (as well as a host of other demographic, social, and economic variables). The analyses transected forty years of population settlement, growth and dispersal. Empirically, we were interested in how Britain’s ethnic geographies had become more complex over time and space. Writing nearly 30 years ago, Robinson (1992) pointed to the *micro*-scale features of immigrant settlement, distinctive even between streets. Our analyses of population grids provided insights into these complex micro-geographies of diversity, and enabled us to take a longer-term perspective than previous studies. We argue that grids provide a unique window into the intricacies of neighbourhood change. Paying attention to the micro-scale is not only analytically richer than a shorter snapshot of change or coarser spatial lens, but it is conceptually useful in attempting to better understand how groups grow and diversify, and spaces change and adapt. Charting change across the whole of Britain offered an additional novelty to previous studies, where Scotland had hitherto received considerably less attention than England and Wales.

Our results can be summarised in four key messages: first, Britain’s ‘ethnic’ diversity (defined by country of birth and ethnic group) has grown steadily over time, and has become more spatially complex, at the finest geographical level. Increases in ethnic diversity have not been constrained to locales that were most diverse in Britain’s recent history; rather, new spaces of diversity have emerged. These fine-grained changes are difficult to appreciate in studies with less spatial or temporal detail, and our approach has thus facilitated a more comprehensive account of these shifting patterns. Our second message is that ethnic group residential segregation at this very small scale has declined for most groups and time points. While in most cases by modest levels, these decreases have tended to be larger by ethnic group than by country of birth, for which there has been a more stable level of segregation. ‘Pioneer’ immigration flows (such as those that are labour-motivated, to specific industrial centres) often pave the way for locational preferences for future immigrations (e.g., joining spouses), reinforcing settlement geographies (Simpson et al. 2008) and maintaining existing concentrations. On the other hand, the dispersal of ethnic minority populations, particularly the UK-born, or those with a longer history in the UK, will have contributed to this decline in ethnic group segregation (e.g., Simpson and Finney 2009). A third observation is that, by the end of our study period, segregation is at its lowest for most ethnic and country of birth groups, and ethnic diversity is at its highest. As Britain’s neighbourhoods have become more diverse, they have become more spatially integrated. Fourth, the most dynamic period of time for changes in ethnic diversity and segregation is the most recent in the dataset: 2001–2011. Our longer time period for analysis enables us to identify the specific period in Britain’s recent history where diversity grew most, and residential segregation decreased most.

While there is much to be offered from the *PopChange* resource, a number of limitations should be recognised. We have emphasised the significance of taking a longer-term perspective on changing ethnic geographies. However, we have only been able to explore five time points for country of birth data, given that the ethnic group question was not introduced until the 1991 British Censuses. Over time, the relationship between country of birth and ethnic group will have weakened. Robinson (1992: 187) recognised that “[birthplace]… is an increasingly poor surrogate for ethnicity because of the large and growing proportions of ethnic minority groups that are now British-born…”. Given the differences in what Census questions on country of birth and ethnic group represent, the geography of diversity is not consistent for these two variables. The former is a measure of country of origin, which may be recent for one person and almost a lifetime ago for another, while the latter captures something more difficult to define, and does not differentiate between place of birth or a heritage outside the UK which may have origins one or more generations ago. Our analysis will not have captured the variation in residential geographies by period of arrival *within* groups, which might be revealing. For example, ONS (2013) show a residential concentration in the West Midlands in 2011 of people with origins in India in the 1960s, whereas those arriving from India during the most recent decade of our study, 2001–2011, were most populous in the north-east and south-west of England, and Wales. Relatedly, the motivations for migration are, of course, also important in determining the geography of settlement. As two examples: the Indian migration of the 2000s just discussed corresponds to a peak in ‘New Commonwealth’ student immigration. Migrants in London were more likely to have come for work or asylum, while migrants who came for family reasons were less likely to live in London (ONS 2013; Blinder and Fernández-Reino 2018; Kone 2018).

The geographies of diversity reflect the processes that shape them, including motivations for immigration, length of time since arrival, internal migration, fertility, and the housing and labour market experiences for ethnic minority groups, which continue to be characterised by marked inequalities and disadvantage (Jivraj and Simpson 2015). The *PopChange* resource offers considerable potential to explore the relationships between diversity and deprivation over time and at a fine spatial scale. More generally, population grids link easily with grid cell data on physical attributes, for example relating to pollution. Population grids also represent opportunities for more meaningful comparisons between countries than where data zones are irregular in their sizes and shapes (e.g., wards or census tracts), and it can be argued that only regular zones (whether grids, hexagons, or any other form of tessellation) are appropriate for inter-country *spatial* comparisons (e.g., see Benassi et al. 2020). However, of course, there are issues of comparability between countries in their measurement of ethnic identity (Mateos 2014). The spatially-detailed chronology of ethnic residential geographies unveiled in this paper also provides opportunities to more accurately inform what is, in the UK as elsewhere, a highly politicised debate on the nature of neighbourhood ethnic concentrations (see Phillips 2007).

## Footnotes

1 *PopChange* website: https://www.qub.ac.uk/research-centres/GIS/Research/PopChange/ and access to the grids via the UK Data Service: https://reshare.ukdataservice.ac.uk/852498/.

2 https://ec.europa.eu/eurostat/web/gisco/geodata/reference-data/population-distribution-demography/geostat.

3 We use the term ‘group’ to reflect that some categories contain multiple countries of birth.
